# P-982. Stewards working smarter not harder: Improving cross-disciplinary communication in bacteremia management

**DOI:** 10.1093/ofid/ofaf695.1181

**Published:** 2026-01-11

**Authors:** Pawlose Ketema, Ayne Adenew, Tiffany Stevens, Erica Carter, Germaine Canzian, Talaya Loran, Munazza Chaudhry, Tina Gonzales, Angela Y McKnight, Angelike P Liappis

**Affiliations:** Veterans Affairs (VA) National Capitol Care Network (VISN5) Antimicrobial Stewardship Clinical Collaborative of VA Medical Centers in Washington DC/Martinsburg WV/Baltimore MD/Clarksburg WV/Beckley WV/Huntington WV, Washington, District of Columbia; DCVA Medical Center, washington, District of Columbia; Washington D.C. VA Medical Center, Washington, District of Columbia; Washington D.C. VA Medical Center, Washington, District of Columbia; Washington D.C. VA Medical Center, Washington, District of Columbia; Washington D.C. VA Medical Center, Washington, District of Columbia; Washington D.C. VA Medical Center, Washington, District of Columbia; Washington D.C. VA Medical Center, Washington, District of Columbia; DC VA Medical Center, Washington, District of Columbia; Washington DC Veterans Affairs Medical Center , Washington, DC

## Abstract

**Background:**

Antimicrobial Stewardship Programs (ASP) respond to real-time data and transmit critical information in patient-level and programmatic interventions across healthcare settings. Our ASP created a sustainable, more efficient workflow for positive blood cultures by leveraging existing electronic platforms to manage real-time data and coordinate clinical management.

**Methods:**

Real-time alerts for positive blood cultures (TheraDoc, DSS Inc) were transferred to a shared secure electronic platform approved for interinstitutional patient-care 5/2022-12/2024 from inpatient, outpatient, long-term care and dialysis units at the Washington DC VA Medical Center. ID Physicians, ID Fellows, Pharmacists and Microbiologists participated in this quality improvement initiative. A PCR array for blood (BioFire, Inc) was introduced onto existing organism identification technology (Sepsityper and MALDI, Bruker Inc) when rapid detection of MDR genes in *E. coli, K. pneumoniae, S. aureus, E. faecalis* might change management. lab posted real-time updates; ASP reviewed therapy and prioritized referrals to ID for serious pathogens or any organism where intensive care required.

**Results:**

487 bacteremic episodes were posted from >1200 + cultures in 321 unique veterans; patients were largely male, older and 1 in 4 experienced >1 episode (98%, 72±13y). Over the period of observation, the continuously maintained secure thread averaged >40 engagement activities (post/reply/reaction) monthly with 15 active users. With preliminary and early results shared directly via thread, stewards advocated for ID consultation and guided therapy choice. By 2024 there was a 60% decrease in PCRs approved (P=0.009) and the acceptance of recommendation to de-escalate based on pre decisional PCR trended higher (30% ↑, P=0.45). Along with other ASP measures and more ID involvement, a trend over time was seen in fewer deaths 7 and 60 days from + culture (50%↓ and 15%↓, P=NS)

**Conclusion:**

The electronic platform promoted active engagement between users and was a sustainable method of cross-disciplinary communication for critical laboratory information. The burden of duplicative efforts was reduced. Stewards prioritized time for consultation, therapy appropriateness, and had more time to communicate with non-ID staff.

**Disclosures:**

All Authors: No reported disclosures

